# Folliculin, the Product of the Birt-Hogg-Dube Tumor Suppressor Gene, Interacts with the Adherens Junction Protein p0071 to Regulate Cell-Cell Adhesion

**DOI:** 10.1371/journal.pone.0047842

**Published:** 2012-11-06

**Authors:** Doug A. Medvetz, Damir Khabibullin, Venkatesh Hariharan, Pat P. Ongusaha, Elena A. Goncharova, Tanja Schlechter, Thomas N. Darling, Ilse Hofmann, Vera P. Krymskaya, James K. Liao, Hayden Huang, Elizabeth P. Henske

**Affiliations:** 1 Division of Pulmonary and Critical Care Medicine, Department of Medicine, Brigham and Women’s Hospital and Harvard Medical School, Boston, Massachusetts, United States of America; 2 Department of Biomedical Engineering, Columbia University, New York, New York, United States of America; 3 Vascular Medicine Research Unit, Brigham & Women’s Hospital and Harvard Medical School, Boston, Massachusetts, United States of America; 4 Pulmonary, Allergy and Critical Care Division, Department of Medicine, University of Pennsylvania, Philadelphia, Pennsylvania, United States of America; 5 Joint Research Division Vascular Biology, Medical Faculty Mannheim and German Cancer Research Center (DKFZ-ZMBH-Alliance), Mannheim, Germany; 6 Department of Dermatology, Uniformed Services University of the Health Sciences, Bethesda, Maryland, United States of America; Baylor University, United States of America

## Abstract

Birt-Hogg-Dube (BHD) is a tumor suppressor gene syndrome associated with fibrofolliculomas, cystic lung disease, and chromophobe renal cell carcinoma. In seeking to elucidate the pathogenesis of BHD, we discovered a physical interaction between folliculin (FLCN), the protein product of the *BHD* gene, and p0071, an armadillo repeat containing protein that localizes to the cytoplasm and to adherens junctions. Adherens junctions are one of the three cell-cell junctions that are essential to the establishment and maintenance of the cellular architecture of all epithelial tissues. Surprisingly, we found that downregulation of FLCN leads to increased cell-cell adhesion in functional cell-based assays and disruption of cell polarity in a three-dimensional lumen-forming assay, both of which are phenocopied by downregulation of p0071. These data indicate that the FLCN-p0071 protein complex is a negative regulator of cell-cell adhesion. We also found that FLCN positively regulates RhoA activity and Rho-associated kinase activity, consistent with the only known function of p0071. Finally, to examine the role of Flcn loss on cell-cell adhesion *in vivo*, we utilized keratin-14 cre-recombinase (K14-cre) to inactivate Flcn in the mouse epidermis. The K14-Cre-Bhd^flox/flox^ mice have striking delays in eyelid opening, wavy fur, hair loss, and epidermal hyperplasia with increased levels of mammalian target of rapamycin complex 1 (mTORC1) activity. These data support a model in which dysregulation of the FLCN-p0071 interaction leads to alterations in cell adhesion, cell polarity, and RhoA signaling, with broad implications for the role of cell-cell adhesion molecules in the pathogenesis of human disease, including emphysema and renal cell carcinoma.

## Introduction

Cell-cell junctions, which include adherens junctions, desmosomes, and tight junctions, are fundamental to the intricate cellular architecture of all epithelial tissues [1]. Because cell-cell junctions allow the epithelium to function as a coordinated tissue, they must be dynamically regulated and remodeled during growth and development, in response to injury, and during normal tissue homeostasis. Adherens junctions link neighboring epithelial cells to the actin cytoskeleton, and desmosomes link them to keratin intermediate filaments. Adherens junctions classically contain E-cadherin and p120-catenin while desmosomes contain desmosomal cadherins and plakophilins. In addition to their structural roles, cell-cell junctions play key roles in cell signaling and are increasingly recognized to participate in cancer pathogenesis [2], [3], [4], [5], [6], [7], [8].

In seeking to elucidate the functions of folliculin (FLCN), the protein product of the Birt-Hogg-Dube (BHD) gene, we used a yeast two-hybrid approach to uncover a physical interaction between the junctional protein p0071 (also called plakophilin 4) and FLCN. p0071 is a relatively understudied member of the armadillo repeat-containing protein family, which includes p120-catenin (p0071’s closest homolog) and beta-catenin [9]. Both p120-catenin and p0071 are components of adherens junctions, and both also localize to the cytoplasm where they regulate the small GTPase RhoA [10]. However, p0071 acts as a positive regulator of RhoA, while p120-catenin is a negative regulator [11], [12].

FLCN is a 64 kDa highly conserved protein with no obvious functional domains. Germline mutations of the *BHD* gene, which is on chromosome 17p11.2 and was cloned in 2002 [13], lead to an autosomal dominant disease associated with fibrofolliculomas (benign skin tumors), cystic lung disease, which can result in spontaneous pneumothorax (lung collapse), and renal cell carcinomas (RCC), which are most often of the chromophobe subtype. The penetrance of these phenotypes is incomplete: 15–30% of BHD patients develop RCC [14], and families have been reported in which cystic lung disease and pneumothorax occur in the absence of renal or skin manifestations [15], [16], [17], [18]. Nearly all germline *BHD* mutations are truncating, and RCC from BHD patients exhibit loss of heterozygosity of chromosome 17p [14], [19], [20], consistent with the hypothesis that BHD is a tumor suppressor gene.

FLCN is known to exist in a complex with AMP-associated protein kinase (AMPK) via two interacting proteins: Folliculin-interacting protein 1 (FNIP1) and FNIP2 [21], [22], [23]. FLCN-deficient cells have dysregulated signaling through AMP-associated protein kinase (AMPK), mammalian target of Rapamycin complex 1 (mTORC1), hypoxia inducible factor (HIF), and transforming growth factor-β (TGF-β) [21], [22], [23], [24], [25], [26], [27], [28], [29], [30]. Despite these advances, the molecular functions of FLCN and the cellular mechanisms through which *BHD* mutations lead to renal and skin tumorigenesis and cystic lung disease are incompletely understood.

We found that downregulation of either FLCN or p0071 results in increased cell-cell adhesion, which is surprising given the conventional view that decreased cell-cell adhesion is associated with tumorigenesis. These results implicate enhanced cell-cell adhesion as a contributing factor in tumorigenesis. To examine this in vivo, we utilized a classic model of cell-cell adhesion integrity: keratin-14 cre-recombinase (K14-cre) [31], [32], [33], which is expressed in the epidermal layer of the skin. K14-Cre-Bhd^flox/flox^ mice have delayed eyelid opening, wavy fur, hair loss, and epidermal hyperplasia, phenotypes that resemble inactivation of other cell adhesion proteins, including p120-catenin, using the same promoter [34]. Together, our data support a model in which FLCN is a guardian of epithelial integrity via its interaction with p0071. Dysregulation of the FLCN-p0071 interaction may underlie the unusual triad of lung, skin, and renal manifestations in BHD patients, and could have critical implications for the pathogenesis of cystic lung disease and chromophobe renal cell carcinoma in the general population.

## Results

### p0071 Interacts with FLCN in Mammalian Cells

**Figure 1:**
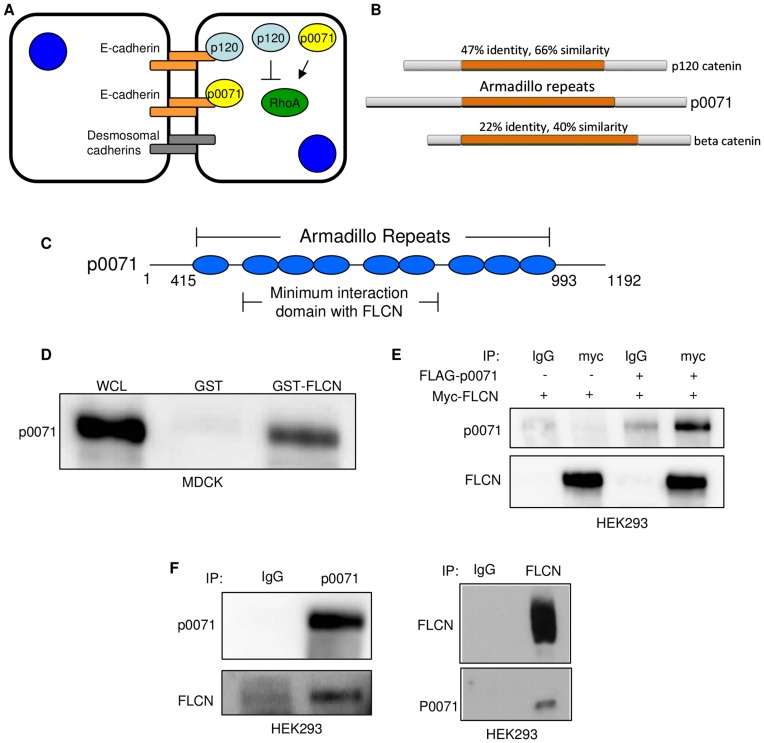
FLCN interacts with p0071. **A)** Schematic of the localization and known functions of p0071 and p120-catenin. **B)** The homology of p0071 compared to the armadillo repeat proteins p120-catenin, the closest homolog of p0071, and beta-catenin. **C)** A representative structure of the armadillo repeats of p0071 (AA 415–993). FLCN interacts with armadillo repeats 2–6 based on the yeast-two-hybrid data. **D)** p0071 interacts with GST-FLCN. MDCK cell lysates were incubated with immobilized GST or GST-FLCN overnight. WCL – whole cell lysate input. **E)** myc-FLCN and FLAG-p0071 or myc-FLCN and FLAG-vector were expressed in HEK293 cells. Myc-FLCN was immunoprecipitated with anti-myc and detected with anti-FLCN. p0071 was detected using anti-p0071 antibodies. IgG antibodies were used as a control (IgG). **F)** Endogenous p0071 immunoprecipitation (left) and FLCN immunoprecipitation (right) in HEK293 cells showing co-immunoprecipitation of FLCN and p0071. IgG antibodies were used as a control (IgG).

To elucidate the molecular functions of FLCN, we used a yeast two-hybrid approach to discover novel FLCN interacting partners. Full-length myc-FLCN was used as bait and screened against a human fetal brain protein library (see Methods). The top hit was p0071 (also called PKP4), which regulates Rho activity [10] and localizes to cell-cell junctions (**Figures 1A–B**). Forty one different prey regions of p0071 interacted with FLCN. Further analysis suggested a minimum interaction domain between amino acids 472–774 of p0071 (**Figure 1C**).

To confirm that FLCN and p0071 interact, we performed a GST-pulldown assay in which Madine-Darby canine kidney (MDCK) cell lysates were incubated with either GST-FLCN, or empty GST. p0071 interacts with GST-FLCN, but not GST (**Figure 1D**). Next, we co-expressed myc-FLCN and FLAG-p0071 in HEK293 cells and performed a myc immunoprecipitation. As shown in **Figure 1E**, FLAG-p0071 co-immunoprecipitates with myc-FLCN. Finally, to determine whether FLCN and p0071 interact at endogenous levels, we immunoprecipitated p0071 or FLCN from HEK293 cells; **Figure 1F** shows that FLCN and p0071 co-immunoprecipitate at endogenous levels. Collectively, these results strongly support a physical interaction between FLCN and p0071.

### FLCN-deficiency Leads to Decreased Rho Activity in Sub-confluent Cells and Defects in Cell Migration

p0071 is known to positively regulate RhoA activity through an interaction with the Rho guanine nucleotide exchange factor, Ect2 [10]. Therefore, to determine whether FLCN-deficiency phenocopies this known function of p0071, we examined Rho-associated kinase (ROCK) activity in FLCN-null UOK257 and FLCN re-expressing UOK257-2 cells. ROCK activity was analyzed by monitoring the phosphorylation status of the myosin-binding subunit of myosin light chain, a downstream substrate of ROCK [35].

**Figure 2:**
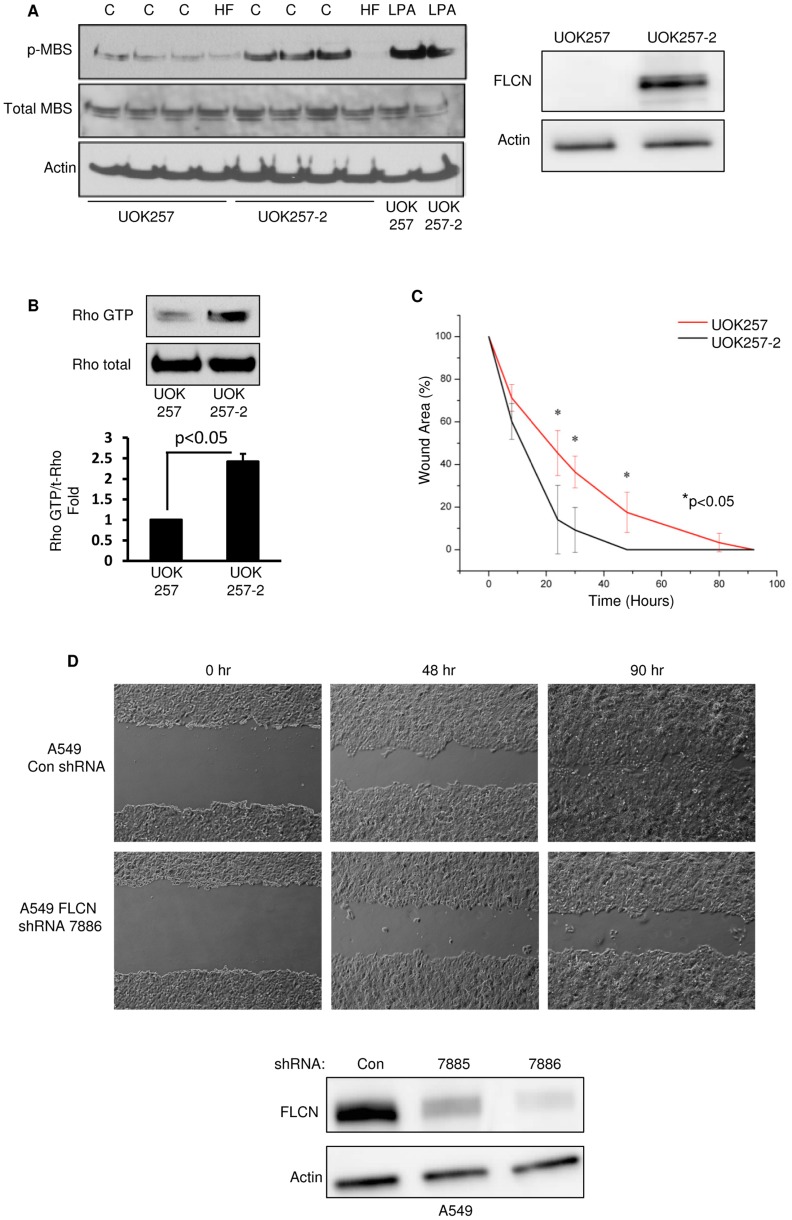
FLCN-deficiency leads to decreased Rho activity in sub-confluent cells and delays in wound closure. **A)** Rho-associated kinase (ROCK) activity was assayed by measuring the phosphorylation of myosin-binding subunit (MBS), a downstream substrate of ROCK. Cells were grown in DMEM supplemented with 10% FBS (C), stimulated with Lysophosphatidic acid (LPA, 30 uM, 30 min), or treated with the ROCK inhibitor Hydroxyfasudil (HF, 20 uM, 30 min). The phosphorylation of MBS was decreased in the FLCN-null UOK257 cells compared to the FLCN-expressing UOK257-2 cells. FLCN levels were analyzed by western blot. **B)** Rho activity was measured using a Rhotekin binding assay at 70% confluence. The FLCN-null UOK257 cells had a 2.5-fold decrease in active Rho levels (Rho GTP) compared to the FLCN re-expressing UOK257-2 cells (n = 3, p<0.05). The data are expressed as mean ± standard error by ANOVA. **C)** A wound assay was used to measure migration in UOK257 cells compared to UOK257-2 cells. A scratch wound was administered (0 hr), and the wound size was measured at 8, 24, 48, 80, and 92 hr post wound induction. At 24 and 48 hours UOK257 cells had a larger wound area than UOK257-2 cells (n = 3, p<0.05) indicating that the FLCN-null UOK257 cells migrate more slowly than the FLCN re-expressing UOK257-2 cells. Data are expressed as mean ± standard deviation. **D)** A scratch wound assay was performed as described in (C) in A549 cells stably expressing FLCN shRNA or control shRNA. Images are shown at wound induction (0 hr), 48 hours post wound induction (48 hr), and 90 hours post wound induction (90 hr). FLCN downregulation was monitored by western blot (bottom).

The phosphorylation of MBS was decreased in FLCN-null UOK257 cells compared to the FLCN-expressing UOK257-2 cells (**Figure 2A:** lanes 1-3 compared to lanes 5-7), suggesting that FLCN positively regulates ROCK activity. Hydroxyfasudil (HF), an inhibitor of ROCK, and Lysophosphatidic acid (LPA), an activator of ROCK, were used as controls. To directly measure RhoA activity we performed a Rho activation assay in 70%-confluent cells. Lysates were incubated with a GST-fusion protein of the Rhotekin-binding domain and active and total Rho levels were analyzed by immunoblot. As shown in **Figure 2B** (top), UOK257-2 cells had a 2.5-fold increase in active GTP-bound Rho compared to UOK257 cells (Figure 2B bottom panel, p<0.05). Finally, we performed a wound-healing assay in UOK257 and UOK257-2 cells and in A549 cells stably expressing control or FLCN shRNA, to determine whether FLCN-deficiency affects cell migration. Both proper RhoA signaling and cell polarity are required for wound closure in vitro. Consistent with the above RhoA data, wound closure in FLCN-deficient UOK257 and A549 cells was delayed when compared to FLCN-expressing cells (**Figure 2C–D**).

### FLCN Negatively Regulates Cell-cell Adhesion and Desmosome Formation

**Figure 3:**
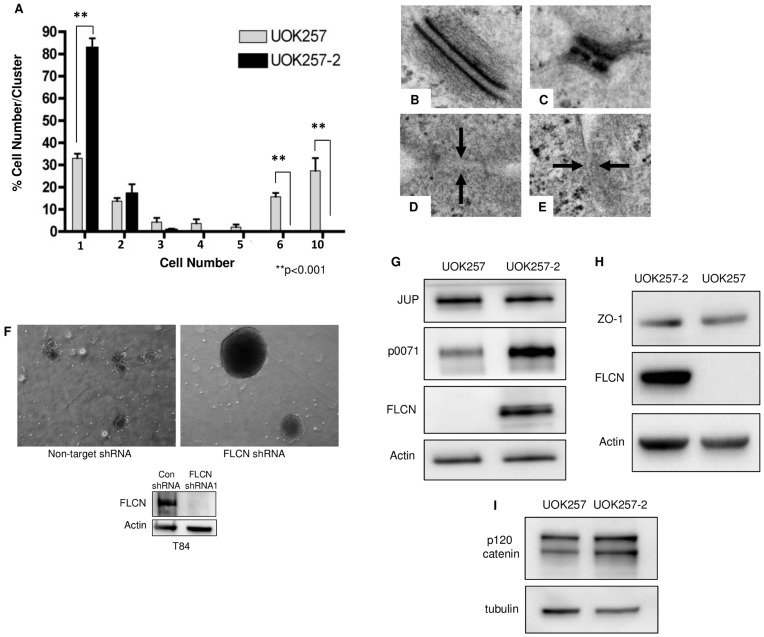
FLCN influences p0071 protein expression, negatively regulates desmosome formation, and negatively regulates cell-cell adhesion. **A)** The hanging drop assay (see Methods for details) was performed in UOK257 and UOK257-2 cells. After shearing, 60% of the FLCN-null UOK257 cells were present in clusters of 6 or more cells. In contrast, none of the FLCN-expressing UOK257-2 cells remained in clusters of 6 or more cells (n = 3, p<0.001) and more than 80% sheared into single cells. (**B–C**) FLCN-null UOK257 cells and FLCN re-expressing UOK257-2 cells (**D–E**) were grown to confluence and analyzed by electron microscopy (50,000×magnification). Desmosomes had normal (B) and abnormal (C) morphology. No desmosomes were seen in the UOK257-2 cells; examples of cell-cell borders (marked by arrows) are shown (D–E). **F)** T84 (colon carcinoma-derived) cells stably expressing either non-targeting (control) or FLCN shRNA were grown in adherence-free conditions (see Methods). FLCN shRNA cells form large, spherical clusters suggesting increased cell-cell adhesion compared to non-targeting control shRNA cells. G–I) p0071, plakoglobin (JUP), ZO-1, and p120 catenin levels were analyzed by western blot in UOK257 cells compared to UOK257-2 cells.

With our discovery that FLCN interacts with p0071 and the known role of p0071 localization to cell-cell junctions, we postulated that FLCN could play a role in cell-cell adhesion. Therefore, we analyzed cell-cell adhesion in UOK257 cells. Cell suspensions were plated as hanging drops on the lid of a tissue culture plate and incubated for three days to form three-dimensional cell clusters; a standard shearing method was then used to disrupt the clusters (see Methods) and the cells were labeled with Hoechst prior to counting. Post shearing, 50% of the FLCN-null UOK257 cells were still observed in clusters of 6 or more cells, while 30% were in groups of ten or more cells (**Figure 3A**); in contrast, none of the FLCN-expressing UOK257-2 cells were found in clusters of 6 or more cells (p<0.001). Furthermore, more than 80% of the sheared FLCN-expressing cells were found as single cells, compared to approximately 30% of the FLCN-null cells (p<0.001). These results suggest that FLCN negatively regulates cell-cell adhesion.

To examine the mechanisms by which FLCN regulates cell-cell adhesion, we next performed electron microscopy on confluent UOK257 (FLCN-null) and UOK257-2 (FLCN-expressing) cells to determine the impact of FLCN-deficiency on cell junction formation. As shown in Figure **3B–E**, FLCN-expressing UOK257-2 cells (D–E) do not form desmosomes; however, FLCN-null UOK257 cells (B–C) do. Interestingly, the FLCN-null cells form both morphologically normal (3B) and abnormal (3C) desmosomes. This data suggests that FLCN negatively regulates desmosome formation, and is of particular interest given that renal carcinomas have increased desmosomes compared to normal kidney tissue [36].

To confirm that the hanging drop results were FLCN-dependent and not cell-type specific to the UOK cells, we grew T84 colon carcinoma cells stably expressing non-targeting or FLCN shRNA in adherence-free conditions using a rotary shaker. T84 cells were chosen because they are often used as an experimental model for studies of cellular junctions [37]. In this assay, cell survival is dependent on the ability of the cells to form clusters. As shown in **Figure 3F**, the FLCN-deficient T84 cells form larger clusters than the control cells. Together with the hanging drop results, these data indicate that FLCN negatively regulates cell-cell adhesion.

### FLCN Influences the Protein Levels of p0071, but not Plakoglobin, ZO-1, or p120 Catenin

To determine whether FLCN is necessary for the expression of cell junction proteins, we measured the levels of p0071, plakoglobin (JUP), zonula occludens-1 (ZO-1), and p120 catenin in UOK257 cells compared to UOK257-2 cells. JUP is a desmosomal and adherens junction protein that forms complexes with catenins and cadherins [38], [39] and ZO-1 localizes to the cell membrane at tight junctions [40], [41]. p120 catenin is an adherens junction protein that is known to regulate Rho activity [42], [43] and is the closest homolog of p0071.

As shown in **Figures 3G–I** **,** p0071 levels are decreased in FLCN-null UOK257 cells, while JUP, ZO-1, and p120 catenin levels are unaltered by FLCN-deficiency.

### p0071 Loss Phenocopies FLCN-deficiency

**Figure 4:**
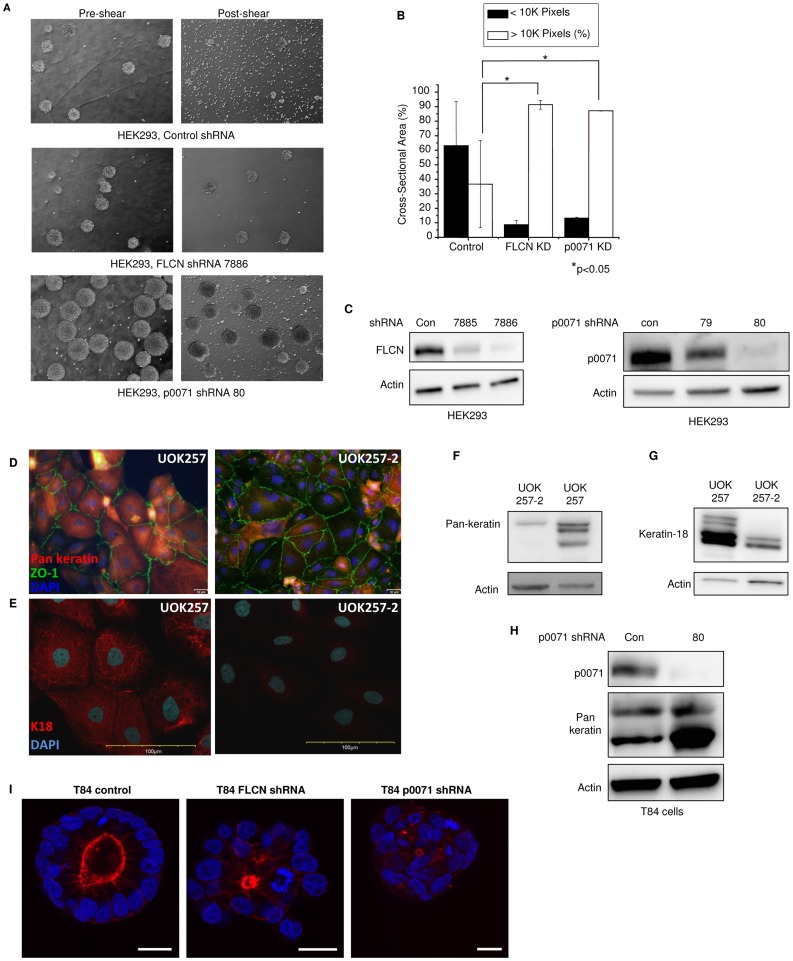
p0071 loss phenocopies FLCN-deficiency. **A)** HEK293 cells expressing control non-targeting shRNA, FLCN shRNA, or p0071 shRNA were cultured in anchorage-independent growth conditions using a rotary shaker and sheared (see Methods for details). A representative phase contrast image is shown pre- and post-shear. **B)** Quantification of cell cluster size from (A) using pixel number. After shearing, 65% of the control cells sheared into clusters of less than 10,000 pixels. In contrast, only 8% (n = 3, p<0.01) of the FLCN shRNA cells and 12% (n = 2, p<0.01) of the p0071 shRNA cells sheared into clusters of less than 10,000 pixels. Data in (A) and (C) are expressed as mean ± standard deviation. **C)** Western blot analysis of HEK293 cells showing decreased levels of p0071 using two different shRNAs (79, 80). Clone 80 was used in (A). **D)** UOK257 cells and UOK257-2 cells were analyzed by immunofluorescence using pan-keratin antibodies (20×magnification). Pan-keratin immunofluorescence was stronger in FLCN-null UOK257 cells. ZO-1 was used to identify cell borders. **E**) UOK257 cells and UOK257-2 cells were analyzed by immunofluorescence using keratin-18 antibodies (60×magnification). Keratin 18 immunofluorescence was also stronger in FLCN-null UOK257 cells. **F)** pan-Keratin levels were compared by immunoblot in UOK257 and UOK257-2 cells. Keratin levels were increased in the FLCN-null UOK257 cells. **G**) Keratin-18 levels were compared by immunoblot in UOK257 and UOK257-2 cells. Keratin-18 levels were also increased in the FLCN-null UOK257 cells. **H)** Keratin levels were analyzed by immunoblot in T84 cells expressing p0071 shRNA (+) or non-targeting control (-) shRNA. Keratin levels were elevated in p0071-deficient T84 cells. **I)** FLCN and p0071 regulate lumen formation in a 3-dimensional matrigel assay. T84 cells stably expressing either non-targeting (control), FLCN, or p0071 shRNA were stained for F-actin (red) and counter stained with DAPI (blue) to visualize nuclei. Downregulation of FLCN or p0071 leads to disorganized, irregularly shaped cell clusters with smaller lumens. The bars represent 20 um.

To determine whether the increased cell-cell adhesion observed in FLCN-deficient cells is mediated via the FLCN-p0071 interaction, HEK293 cells that stably express non-targeting, FLCN, or p0071 shRNA were grown in adherence-free conditions as in 3F. After a standardized shearing protocol (see Methods) the HEK293 cells expressing control shRNA sheared into single cells or small clusters (**Figure 4A** top right panel vs. top left panel). In contrast, the HEK293 cells expressing FLCN shRNA remained in large clusters after shearing (Figure 4A middle right panel vs. middle left panel). Interestingly, in this assay, cells expressing p0071 shRNA also formed large shear-resistant clusters (compare Figure 4A bottom right and bottom left panels), similar to those formed by the FLCN shRNA-expressing cells. Quantification of these findings revealed that after shearing, 65% of the control cells formed clusters of less than 10,000 pixels, whereas only 8% of the cells that expressed FLCN shRNA (p<0.05) and only 12% of the cells that express p0071 shRNA cells (p<0.05) formed clusters of less than 10,000 pixels (**Figure 4B**). Downregulation of FLCN and p0071 is shown in **Figure 4C**. These data suggest that FLCN regulates cell-cell adhesion through its interaction with p0071.

Keratins are the major structural component of intermediate filaments in desmosomes, and play a critical role in cell-cell and cell-matrix adhesion [44], [45]. Our discovery that FLCN negatively regulates desmosome formation (Fig 3B–E) led us to hypothesize that keratin levels are affected by FLCN-deficiency. To explore this, we performed pan-keratin immunocytochemistry on UOK257 cells compared to UOK257-2 cells. As shown in **Figure 4D**, the keratin immunofluorescence was stronger in UOK257 cells compared to UOK257-2 cells at the same exposure. Interestingly, the keratin intermediate filaments appear to be diffusely localized in the UOK257 and UOK257-2 cells. Additionally, we analyzed keratin-18 immunofluorescence via confocal microscopy. As shown in Figure 4E, keratin-18 is more intense in UOK257 cells compared to UOK257-2 cells. To verify that keratins are increased at the protein level, we analyzed pan-keratin (Figure 4F) and keratin-18 (Figure 4G) by western blot in the UOK cells. UOK257 cells displayed increased pan-keratin and keratin-18 protein levels compared to UOK257-2 cells, indicating that FLCN-deficiency leads to increased keratin levels consistent with our finding that FLCN regulates desmosome formation. Downregulation of p0071 also led to increased levels of keratins (**Figure 4H**) suggesting that keratin levels are mediated via the FLCN-p0071 interaction.

### p0071 and FLCN Impact Three Dimensional Lumen Formation

The establishment of proper apical-basal polarity is another important component of cellular homeostasis and defects in cell polarity are commonly observed in tumorigenesis. Therefore, we next examined the impact of FLCN and p0071 downregulation on cell polarity. T84 cells stably expressing either non-targeting, FLCN, or p0071 shRNA were grown in mixed extracellular matrix proteins. These 3-dimensional matrices cause epithelial cells to form spherical cysts consisting of a monolayer of epithelial cells around a hollow lumen. The individual cells are connected to each other by cell-cell contacts and form a highly polarized structure where the apical surface is oriented to the lumen of the cyst [46]. As shown in **Figure 4I**, downregulation of FLCN or p0071 led to irregularly shaped and less organized cyst formation with decreased lumen size. Taken together with our cell adhesion data, these data suggest that **deficiency of either FLCN or p0071** leads to increased cell-cell adhesion and defects in cell polarity.

### Conditional Inactivation of *Bhd* in the Mouse Epidermis Leads to Hair and Skin Abnormalities

**Figure 5:**
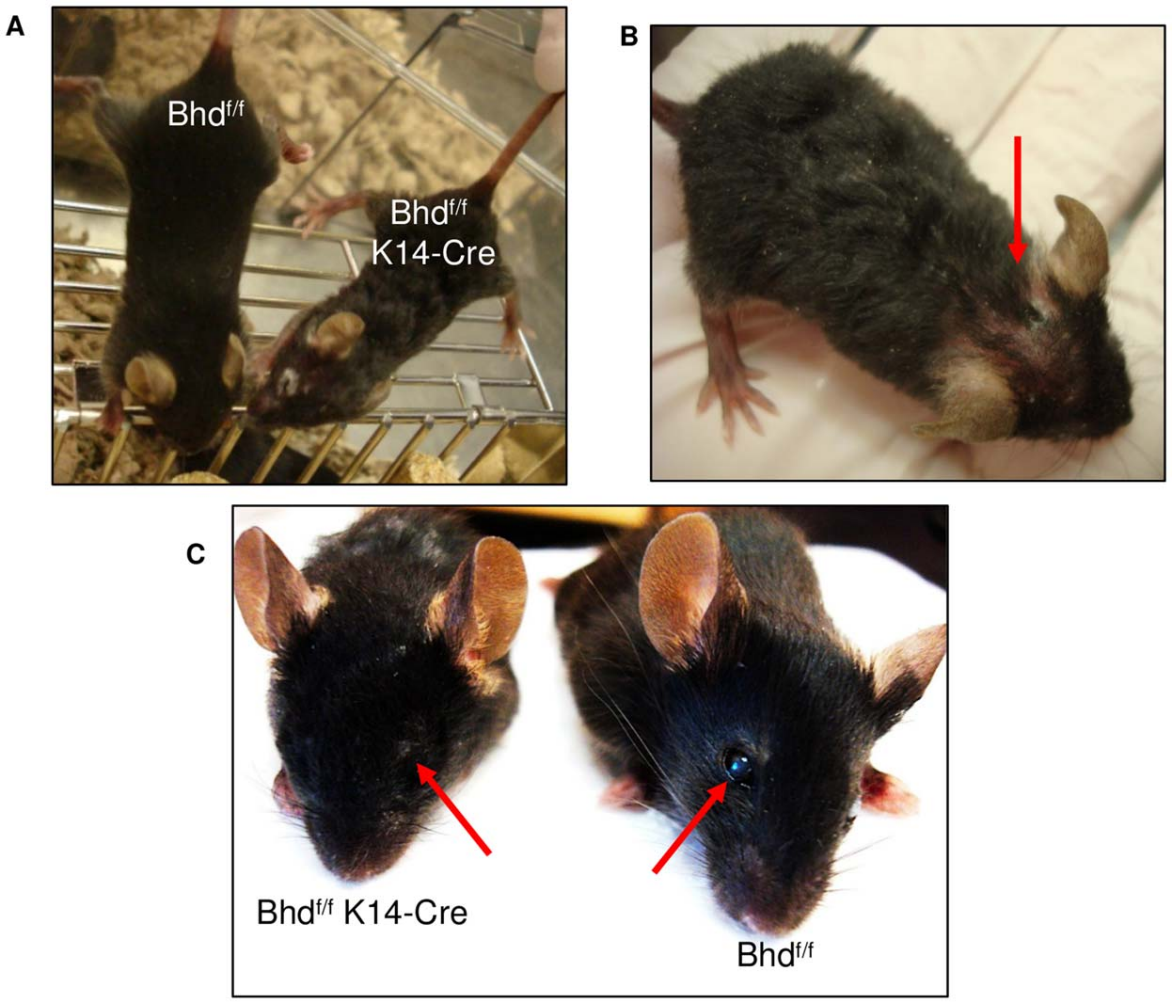
Bhd^f/f^ K14-Cre mice have wavy hair, erythematous skin, and delayed eyelid opening. **A)** Bhd^f/f^ K14-cre mice at three weeks of age display coarse, wavy fur and wavy whiskers compared to Bhd^f/f^ wild-type mice. **B)** A representative Bhd^f/f^ K14-cre mouse at three weeks of age with erythematous skin and hair loss (arrow). **C)** 100% of Bhd^f/f^ K14-cre mice have closed eyelids at three weeks of age (arrow) compared to 0% of wild-type mice.

Inactivation of junctional proteins with keratin-14 driven cre-recombinase (K14-cre) is used frequently to study cell-cell adhesion *in vivo* [31], [32], [33]. K14-cre is expressed in the skin epithelium beginning at E13.5 [47], [48]. We used K14-cre to conditionally inactivate *Bhd* in the mouse skin, in order to evaluate the effects of FLCN-deficiency on cell adhesion *in vivo*. Bhd^flox/flox^ (Bhd^f/f^) mice [49] were crossed with K14-cre mice to generate Bhd^f/f^ K14-cre mice. By three weeks of age, the Bhd^f/f^ K14-cre mice were significantly smaller than their littermate controls, developed coarse and wavy hair, and displayed erythematous patches of skin and hair loss, which progressed with age (**Figure 5A–B**). In addition, 100% of the littermate wild type control mice had opened their eyes by two weeks of age, but none of the Bhd^f/f^ K14-cre mice had done so (representative image shown in **Figure 5C** **)**.

**Figure 6:**
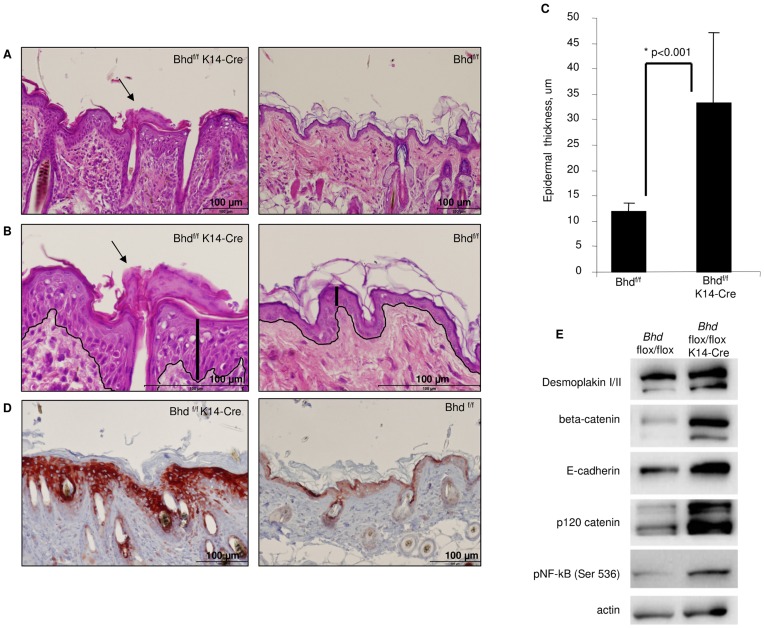
Bhd^f/f^ K14-Cre mice exhibit increased epidermal thickness. A) Bhd^f/f^ K14-cre mice exhibit acanthosis and hyperkeratosis (arrows) compared to littermate control Bhd^f/f^ wild-type mice at three weeks of age. H&E stain, 20×magnification. **B)** Skin region from (A) at 40×magnification with the epidermal layer outlined and vertical bars showing the thickness from the epidermal basement membrane to the granular layer. Scale bars are 100 µm for both (A) and (B). **C)** Quantification of the thickness of the epidermis. The Bhd^f/f^ K14-cre mice have a 3-fold increase in thickness of the epidermis (n = 4, p<0.001). Data are expressed as mean ± standard deviation. **D)** Bhd^f/f^ K14-cre mice exhibit elevated levels of phosphorylated ribosomal protein S6 (S235/236, red staining), a readout or mTORC1 activity, compared to littermate control Bhd^f/f^ wild-type mice at three weeks of age. 20×magnification. E) A representative western blot of the skin of a 7 month old Bhd^f/f^ K14-cre mouse compared to the skin of a littermate control at 7 months of age. beta-catenin, E-cadherin, p120 catenin, and phospho-NF-kB levels are increased in the mutant skin.

Further analysis of the Bhd^f/f^ K14-cre mouse skin revealed a thickened epidermis involving the spinous layer (acanthosis), granular layer (hypergranulosis) and stratum corneum (hyperkeratosis) (**Figure 6**). In the epidermis, the distance from the basal layer to the granular layer was 3-fold greater compared to littermate controls (p<0.001), and displayed focal areas of hyperkeratosis. Lastly, Bhd^f/f^ K14-cre mice display elevated levels of phosphorylated ribosomal protein S6, an indicator of mTORC1 activity, in the hyperproliferative epidermis, suggesting that FLCN negatively regulates mTORC1 in the skin.

## Discussion

BHD is an autosomal dominant disease associated with lung cysts, skin tumors, and kidney cancer, primarily of the chromophobe subtype [50], [51], [52], [53]. Germline *BHD* mutations also occur in families with hereditary lung collapse (pneumothorax) without renal or skin disease [15], [16], [17], [18]. Somatic *BHD* mutations occur in ∼10% of sporadic chromophobe RCC [54]. We report here that FLCN interacts with the adherens junction protein p0071 to regulate cell-cell adhesion, cell migration, cell polarity, and RhoA signaling. Furthermore, inactivation of FLCN in the basal layer of the epidermis, which is classically used to study the in vivo impact of cell adhesion proteins on the integrity of epithelial architecture, results in striking phenotypes including wavy fur, epidermal hyperplasia and inflammation, and defective eyelid opening.

These data provide a critical new component to our emerging understanding of the pathogenesis of BHD. BHD is one of the least studied monogenic tumor suppressor gene disorders, with fewer than 12 publications on the functions of FLCN. FLCN has been linked to AMPK signaling via a direct interaction with FNIP1/2, to HIF signaling and mitochondrial dysfunction, to aberrant TGF-beta signaling, and to mTOR complex 1 signaling [21], [22], [23], [25], [29], [49], [55], [56], [57], [58], [59], but the fundamental mechanisms through which loss of FLCN leads to abnormalities in three epithelial organs, the lung, the kidney, and the skin, remain incompletely understood. Our data point toward a completely new model in which regulation of the integrity of the epithelium via cell-cell junctions is a critical function of FLCN.

Our finding that FLCN-deficient and p0071-deficient cells have enhanced cell-cell adhesion is unexpected, given that cell-cell junctions are critical for epithelial tissue homeostasis and loss of the integrity of these junctions is associated with cancer progression [2], [5], [7]. p120-catenin, the closest homolog of p0071, localizes to adherens junctions and positively regulates cell adhesion in prostate cancer-derived [60] and lung cancer-derived cells [61]. p0071 (also called PKP4) localizes to adherens junctions, but its function in cell-cell adhesion has not been previously studied. Additionally, there is controversy regarding the desmosomal localization of p0071. We have observed desmosomal defects in FLCN-deficient cells (Figure 3), but at this stage we cannot ascribe the FLCN effects exclusively to one type of junction. Furthermore, the effects of loss of FLCN or p0071 may result in part from the impact on p0071’s other interacting partners, which include E-cadherin [62], a critical component of adherens junctions. Germline loss-of-function mutations in the *CDH1* gene, which encodes E-cadherin, cause human gastric cancer [3], [4], [6], [8].

In addition to their localization to adherens junctions, both p120-catenin and p0071 localize to the cytoplasm where they regulate RhoA activity [10], [11], [12]. Our data that FLCN-deficient cells have low levels of RhoA and ROCK activity are consistent with the known role of p0071 as a positive regulator of RhoA, but surprising given that RhoA is often activated in human cancer [63], [64], [65]. The TGF-beta signaling network, which is often activated in human cancer [66], [67], [68], is also downregulated in FLCN-deficient cells, and mTORC1 signaling can be either up or downregulated in FLCN-deficient cells depending on the cell type and context [21], [25], [49], [55], [56]. Therefore the signaling consequences of FLCN deficient cells do not appear to align precisely with those of other tumors, or of other genetic diseases associated with kidney cancer and skin tumors, such as tuberous sclerosis complex [69], [70], [71]. Some of the signaling functions of FLCN for which a molecular mechanism is not yet clearly identified, including mTORC1 and TGF-beta, could be downstream of the FLCN-p0071 interaction, either through a mechanism arising at the cell-cell junction or via RhoA.

Patients with mutations in the *BHD* gene develop renal cell carcinomas (RCC), the majority of which are chromophobe and chromophobe/oncocytic hybrid tumors, and somatic *BHD* mutations have been found in sporadic chromophobe RCC, which is a rare disease affecting ∼1500 individuals in the United States each year. There appear to be at least two potential links between defective cell junctions and sporadic RCC. First, we found that FLCN-deficient cells have more desmosomes than FLCN-expressing cells, and Kim et al. found that desmosomes are increased in RCC compared to normal kidney tissue and identified an association between desmosome number and tumor grade [36]. Second, Walter et al. found that p0071, which is expressed in human and mouse kidney epithelium [7], [72], appears to be lost in 41% of chromophobe RCC [7]; we found that p0071 interacts with FLCN and that loss of p0071 phenocopies loss of FLCN in cell-based assays. These fundamental mechanistic connections between cell-cell junctions and the pathogenesis of sporadic chromophobe RCC could eventually lead to an improved understanding of disease pathogenesis and the development of targeted strategies for metastatic chromophobe RCC, for which there are currently no known effective therapies.

The cellular and physiologic mechanisms that lead to cystic and emphysematous airspace enlargement in BHD are not known. Respiration exposes the lung to unique mechanical forces. Cell-cell junctions allow the lung to expand in response to these forces and then “snap back” to their original shape and organization. Mechanical-induced stretch of pulmonary alveolar type II cells leads to widened cell-cell gaps and is speculated to be a protective mechanism in response to lung injury, likely due to a flexibility effect [73], and defects in tight junctions lead to lung injury and fibrosis in mice [74]. We hypothesize that the defects in cell-cell adhesion in FLCN-deficient cells underlie the pathogenesis of airspace enlargement in BHD. If correct, this could lead to an improved understanding of airspace enlargement in other diseases including emphysema and chronic obstructive pulmonary disease (COPD), the third leading cause of death in the world.

Finally, we have developed a novel mouse model in which *Bhd* is inactivated in the epidermis via the Keratin-14 (K14) promoter. K14-cre driven inactivation of adhesion proteins has been used to study other junctional proteins *in vivo* [31], [34], [75]. Most notably, K14-Cre driven inactivation of p120-catenin, the closest homolog of p0071, leads to epidermal hyperplasia and hair loss [34] that resemble those of FLCN inactivation. Rho signaling is known to regulate keratinocyte differentiation and proliferation [76], [77], and may contribute to the epidermal consequences of p120-catenin and FLCN inactivation.

**Figure 7:**
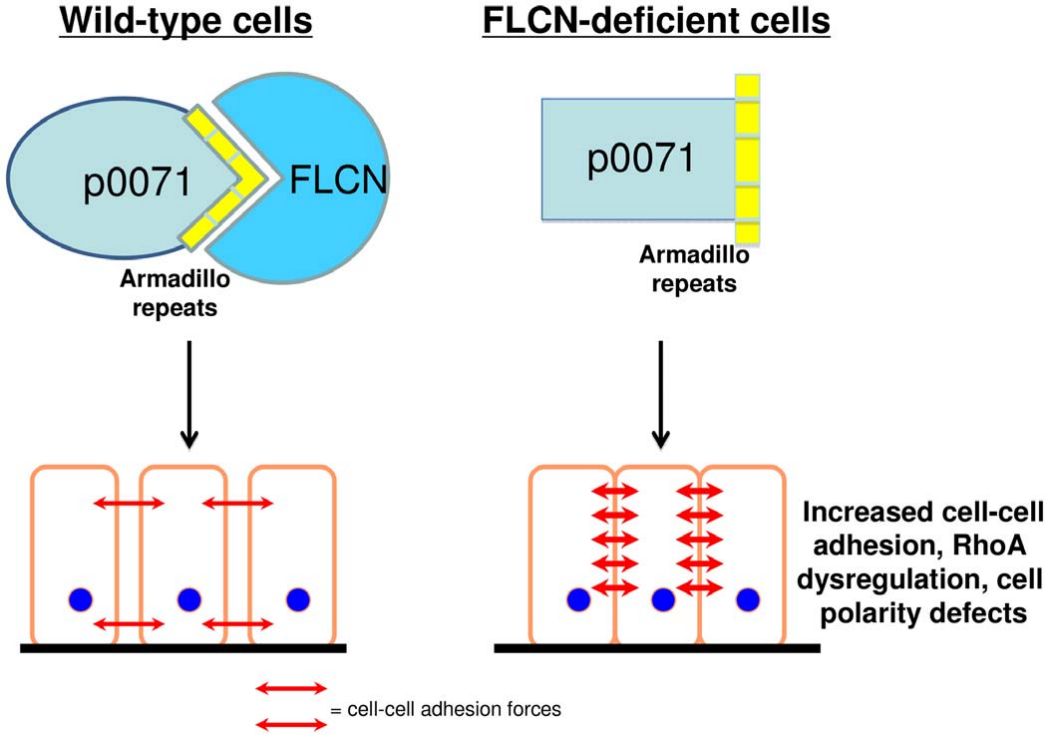
Working Model. In wild-type cells (left) the FLCN-p0071 interaction is required for maintenance of normal epithelial cell-cell adhesion and proper cell polarity. Red arrows indicate cell-cell adhesion forces. In FLCN-deficient cells (right), loss of the FLCN-p0071 interaction leads to enhanced cell-cell adhesion, though we do not currently understand which cellular junctions are critical for this defect, and is accompanied by defects in RhoA signaling and cell polarity.

In conclusion, we have uncovered a physical interaction between FLCN and p0071 that is critical for the proper regulation of cell-cell adhesion, RhoA activity, and epithelial cell polarity. A mouse model of FLCN inactivation in the epidermis leads to wavy hair and whiskers, delays in eyelid opening, hair loss, and increased epidermal thickness, phenotypes which resemble the inactivation of p120-catenin, the closest homolog of p0071. Taken together, these findings reveal a novel paradigm in which loss of the FLCN-p0071 interaction and consequent defects in cell-cell adhesion, cell polarity and RhoA activity participates in the pathogenesis of renal cell carcinoma, skin tumors, and cystic airspace enlargement (Figure 7).

## Materials and Methods

### Yeast Two-hybrid Assay

The yeast two-hybrid assay was performed by Hybrigenics (Paris, France). Full-length human FLCN coding sequence was PCR amplified and inserted into pB29 vector as an N-terminal fusion to LexA (N-FLCN-LexA-C). The construct was used as a bait to screen the Hybrigenics Human Fetal Brain_RP1 library. The DNA insert of the positive clones was sequenced to identify the corresponding gene in the GenBank database.

### In vitro Binding Assay

Human BHD cDNA was cloned into the pGEX4T-2 expression vector (GE Healthcare) and transformed into BL-21 bacteria (Stratagene). Isolation and purification of GST-FLCN or empty GST control was performed according to a previously published protocol [78]. Isolated recombinant proteins were immobilized on glutathione agarose and incubated with MDCK lysates overnight. GST-FLCN or empty GST beads were washed three times with lysis buffer and boiled. The interacting proteins were separated by SDS-PAGE and analyzed by immunoblot.

### Cell Culture, Transfections and Plasmids

The T84, HEK293 and MDCK cell lines were purchased from ATCC; FLCN-null UOK257 cells and FLCN re-expressing UOK257-2 cells were a gift from Drs. Laura Schmidt and Marston Linehan (National Cancer Institute, Bethesda, MD) [79]. All cells were maintained in DMEM supplemented with 10% FBS and 1% pen/strep; the UOK257-2 cells were additionally supplemented with 2 ug/mL blasticidin S (Invitrogen). Transient DNA transfections were carried out using Fugene 6 (Roche). The myc-FLCN expressing plasmid has been described previously [25] and the FLAG-p0071 plasmid was a gift from Dr. Andrew Kowalczyk at Emory University [80].

### Immunoblotting

Cells were lysed on ice in urea lysis buffer (9 M urea, 1% NP-40, 25 mM Tris pH 7.4, and 150 mM NaCl) and centrifuged at 14,000 rpm for 15 minutes. The supernatants were boiled, proteins separated by SDS-PAGE, and transferred onto PVDF membrane. Membranes were incubated with the following antibodies: anti-FLCN, anti-zonula occludens-1, anti-pan-keratin, and anti-keratin-18 (Cell Signaling Technology); anti-actin, anti-JUP, and anti-tubulin (Sigma); and anti-p0071 (Progen Biotechnik, Germany); and anti-p120-catenin (Life Technologies). Chemiluminescent signals were captured using a Syngene G-BOX iChemi XT imager and quantified with Syngene GeneTools software.

### Hanging Drop Assay

Cell aggregates were prepared using a previously described approach [81]. Briefly, 4000 cells suspended in a 20 uL drop were pipetted onto the sterile cover of a culture dish. After 4 days, cell aggregates were harvested and transferred to a tube. To shear, Hoechst was added to each cell suspension, incubated at 37°C for 10 minutes, and pipetted 100 times in order to fragment the cell aggregates. Bright field images were acquired using an Olympus IX-81 microscope under 4×magnification.

### Cell Aggregate (Rotary Shaker) Assay

100,000 HEK293 cells were seeded on a 60 mm suspension culture dish. Cells were incubated at 37°C on an orbital shaker at 70 rpm for 4 days, and allowed to form aggregates. Aggregate suspensions were harvested and sheared by pipetting 15 times using a P200 pipet. The sheared cell suspension was then transferred to a 35 mm dish and imaged under bright field using an Olympus IX-81 microscope. Aggregate size was quantified using ImageJ and the Integrated Morphometry Analysis feature in Metamorph (Sunnyvale, CA).

### 3D epithelial Cell Culture

T84 cells with stable knock down (FLCN, p0071 or control) were maintained in DMEM supplemented with 10% FBS and Puromycin (5 ug/mL). For 3D culture, approximately 4.5×10^3^ cells were trypsinized to a single cell suspension and re-suspended in 500 uL medium with 2.5% Matrigel (BD Biosciences, Heidelberg, Germany) and plated on coverglass chambers (8 well chamber slides, Nunc) precoated with 5 uL of 100% Matrigel. Cysts were allowed to form over 5–7 days at 37°C. For visualization of F-actin with phalloidin Alexa 546 (Molecular Probes, Invitrogen, Karlsruhe, Germany) the fixation and staining procedure of Chu et al. was followed [82]. Labeled cells counterstained with DAPI were examined using a Zeiss LSM 700 laser scanning confocal microscope. Images demonstrate a single confocal section acquired at the center of the cyst.

### Wound Healing Assay

Cells were plated at 75% confluency in three 35 mm dishes, and cultured until fully confluent. Linear wounds were created by scraping a P200 pipette tips across the surface of each confluent monolayer. Bright field images of wound migration were acquired at 0, 8, 24, 48 80 and 92 hours at 4×magnification using an Olympus IX-81 microscope. For each time point, the wound size in pixels was calculated for each dish, and the average of the 3 dishes was used as a single data point.

### ROCK Activity Assay

The phosphorylation of the myosin binding subunit (MBS) was measured to analyze Rho-associated kinase (ROCK) activity using a modification from a previously published method [35]. Briefly, cells were harvested and fixed on ice in trichloroacetic acid (50%, Sigma) and dithiothreitol (50 mM, Sigma) dissolved in dH2O (freshly made before fixation). Cells were pelleted by centrifugation at 4°C. The pellet was washed 2 times with PBS and resuspended in extraction buffer (8 M urea, 20 mM Hepes pH8). Cell extracts were subjected to 7% SDS-PAGE and transferred onto PVDF membrane. Membranes were incubated with rabbit anti-phospho-specific Thr853-MBS polyclonal antibody [83] or rabbit anti-MBS polyclonal antibody (Covance). ROCK activity was expressed as the ratio of phospho-MBS/total MBS.

### Rho Activity Assay

Rho activity was examined using the Active Rho Pull-Down and Detection Kit (Pierce Biotechnology) as previously described [84]. Cells were serum-deprived for 16 h and lysed in Lysis/Binding/Wash buffer. Lysates were incubated with a GST-fusion protein of the Rhotekin-binding domain (RBD) and glutathione agarose resin for 1 hour in order to pull-down Rho-GTP. The resin was washed and Rho was extracted with 2×SDS sample buffer followed by Western blot analysis with anti-Rho antibody. The data are presented as Rho-GTP/total Rho ratio and are mean values ± SE by ANOVA (Fisher test).

### Generation and Analysis of Mice

All mouse experiments were carried out under guidelines approved by the ARCH Institutional Animal Care and Use Committee (IACUC). The ARCH IACUC specifically approved this study. Transgenic mice with skin-specific inactivation of mouse *Bhd* were generated by crossing Bhd^f/f^ mice (a gift from Drs. Laura Schmidt and Marston Linehan) with Keratin14-Cre transgenic mice (Jackson Laboratories) to establish Bhd^f/+^ K14-Cre animals. Bhd^f/+^ K14-Cre mice were intercrossed to generate Bhd^f/f^ K14-Cre mice. Tissue processing and staining was performed by the Rodent Histopathology Core (Harvard Medical School). Epidermal thickness was measured using Olympus FSX100 integrated software.
